# Hypothermic Machine Perfusion Preservation of the DCD Kidney: Machine Effects

**DOI:** 10.1155/2013/802618

**Published:** 2013-10-10

**Authors:** Susanne L. Lindell, Heather Muir, John Brassil, Martin J. Mangino

**Affiliations:** ^1^Departments of Surgery, Virginia Commonwealth University, Medical College of Virginia Campus, Richmond, VA 23298-0454, USA; ^2^Functional Circulation, Northbrook, IL 60062, USA; ^3^Emergency Medicine, Virginia Commonwealth University, Medical College of Virginia Campus, Richmond, VA 23298-0454, USA; ^4^Physiology and Biophysics, Virginia Commonwealth University, Medical College of Virginia Campus, Richmond, VA 23298-0454, USA

## Abstract

*Purpose*. Kidneys from DCD donors represent a significant pool, but preservation problems exist. The study objective was to test the importance of machine type for hypothermic preservation of DCD kidneys. *Methods*. Adult Beagle dog kidneys underwent 45 minutes of warm in situ ischemia followed by hypothermic perfusion for 24 hours (Belzer-MPS Solution) on either an ORS LifePort or a Waters RM3 using standard perfusion protocols. Kidneys were then autotransplanted, and renal function was assessed over 7 days following contralateral nephrectomy. *Results*. Renal vascular resistance was not different between the two pumps. After 24 hours, the oxygen partial pressure and oxygen delivery in the LifePort perfusate were significantly lower than those in the RM3 but not low enough to change lactate production. TheLifePort ran significantly colder than RM3 (2° versus 5°C). The arterial pressure waveform of the RM3 was qualitatively different from the waveform of the LifePort. Preservation injury after transplantation was not different between the devices. When the LifePort was changed to nonpulsatile flow, kidneys displayed significantly greater preservation injury compared to RM3. *Conclusions*. Both LifePort and RM3 can be used for hypothermic machine perfusion preservation of DCD kidneys with equal outcomes as long as the duty cycle remains pulsatile.

## 1. Introduction

Renal transplantation continues to be the treatment of choice for patients with end stage renal disease. Currently, over 91,000 renal patients are wait listed to receive a kidney transplant while last year only 17% were transplanted [1]. While the wait list for kidneys seems to grow geometrically, the rate of transplantation grows linearly, leaving centers to look for new ways to expand the donor pool.

One source of kidney donors that have gained considerable attention over the last decade is Donation after Cardiac Death (DCD). The pool of controlled DCD kidneys is small since only about 10% of kidneys come from this category [2]. The pool for uncontrolled DCD kidney donors, however, is potentially huge. It has been estimated that of the 335,000 cardiac deaths that occurred in 2006, at least 22,000 (7%) may meet the criteria for uncontrolled DCD donation [3]. This has the conservative potential of unleashing 44,000 kidneys per year into the donor pool, which could seriously alleviate the donor shortage since the total number of all deceased donor kidney transplants in the USA in all of 2011 was only 11,043. Furthermore, the technical and logistic ability to use these kidneys is high in large cities that share both a major transplant center and a busy Emergency Medicine/Trauma service, thus making the theoretical implications for using uncontrolled DCD kidneys more realistic. However, the problem with using uncontrolled DCD kidneys is the necessary exposure of warm ischemia to the grafts before harvest and preservation. While the effects of 30–90 minutes of warm renal ischemia per se produce measured reperfusion injury to the kidney at transplantation, the addition of inevitable periods of hypothermia from traditional organ preservation, no matter how brief, produces potentiating injury that exceeds the sum of both factors combined [4, 5]. This typically results in very high incidence of delayed graft function (DGF) following transplantation [6].

To mitigate against heightened preservation injury in DCD kidneys, hypothermic pulsatile machine perfusion preservation is utilized. The theoretical benefits of perfusion of DCD kidneys have been recently demonstrated conclusively. Canine kidneys with 60 minutes of prior warm ischemia were 100% viable when perfused with UW-based solutions on a Belzer pulsatile perfusion machine for 24 hours compared to 40% viable when cold stored in UW solution for the same time [4]. Similar results have recently been observed in clinical studies. Moers demonstrated an overall superiority of perfusion preservation, relative to simple cold storage, of kidneys in a paired prospective large clinical trial. Graft viability of paired donor kidneys was significantly improved when one kidney was machine perfused compared to the cold stored contralateral control kidney after 1 year [7] or 3 years after transplantation [8]. In the 3-year study [8], the machine perfusion effect was dramatically amplified in a subgroup of patients that experienced initial delayed graft function. Finally, hypothermic machine perfusion has recently been demonstrated to be superior to cold storage in preservation of kidneys from DCD donors [9]. This further supports the utility of using machine perfusion in reanimating injured kidneys from DCD donors. By significantly reducing the incidence of DGF in this donor group [9], hypothermic machine perfusion should also improve the long term half-life of these grafts. 

Today, hypothermic machine perfusion of kidneys in the transplant clinic typically utilizes one of two types of perfusion machines: the Waters (Waters Medical Systems) RM3 or the ORS (Organ Recovery Systems) LifePort. Although both machines are designed to perfuse kidneys with cold crystalloid preservatives under pulsatile conditions, many engineering and performance differences raise the possibility that their performance under DCD conditions is also different. Such differences include perfusate oxygenation, perfusate operational temperatures, motor duty cycle, arterial waveform differences, and differences in how they are typically used by clinicians (operational protocols). Characterizing outcomes between these two devices in a prospective controlled pre-clinical setting was the objective of this study since it has never been done. The hypothesis was that kidneys from DCD donors would perform better after preservation on the Waters RM3 compared to the ORS LifePort because of the availability of oxygen and the pulsatile duty cycle of the pump. 

## 2. Materials and Methods

### 2.1. Materials and Methods

Adult Beagle dogs were anesthetized with propofol (10–17 mg/kg, I.V. bolus) followed by intubation and isoflurane inhalation anesthesia at 1–3% with 50% oxygen. All animal studies were conducted under a protocol approved by the VCU IACUC. After anesthesia, a midline incision was made to expose the left kidney. The vessels and ureter were isolated and skeletonized. Both the renal artery and vein were ligated with 0-silk ties for 45 minutes to induce warm renal ischemia. After the ischemic period, the kidney was removed and the renal artery was quickly flushed with about 100 mL of cold saline containing Heparin (5,000 U/L). After organ donation, the animals were closed and recovered from surgery using standard techniques. The kidneys were immediately placed on a perfusion machine for 24 hours. Each kidney was randomized to either the RM3 or the LifePort, and 8 dogs were used for each treatment arm. In a subgroup of 4 additional dogs, the perfusate was delivered without pulsations (at 30 mm Hg) on the LifePort. Each group was perfused with cold Belzer MPS solution according to the following protocols that are typically used in the clinic. 

#### 2.1.1. LifePort

The kidneys were perfused from the start at a mean pressure setting of 30 mm Hg in pulsatile mode. Four additional dogs were perfused in nonpulsatile mode. The LifePort actively maintains this pressure by continuously adjusting the flow rate by feedback. 

#### 2.1.2. RM3

The kidneys were perfused at an initial flow that produced a peak systolic pressure of 45 mm Hg by slowly raising the flow rate over 10 minutes. Then the flow rate was continuously adjusted to maintain this target systolic pressure over the next 4 hours. After 4 hours, the pump flow rate was held constant for the next 24 hours and the perfusion pressure was allowed to change in step with changes in resistance. 

The LifePort was cooled by an internal heat exchanger using melting ice while sitting on the lab bench while the RM3 was cooled by placing the entire unit in a cold room, which was actively maintained at 6–8°C. The RM3 unit was oxygenated by sweeping air over the membrane oxygenator at about 2–4 L/min while the LifePort had no active or passive method of perfusate oxygenation. Performance of the kidneys on the pump was monitored by recording perfusion pressure, perfusate flow, vascular resistance, perfusate temperature, pH, electrolytes, and pO_2_ during the perfusion period. After 24 hours of perfusion preservation, kidneys were removed from the pump and autotransplanted into the original donor animal as previously described [4]. At the time of autotransplantation, the right contralateral kidney was removed. No post-operative immunosuppressive medications were required or given. Renal function and preservation injury were assessed each post-operative day by measuring renal function by a daily venous blood draw. Serum creatinine was measured in daily venous blood samples for 7 days using a Vet Scan HM5 (Abaxis, Union City, CA) clinical chemistry analyzer. After the 7-day observation period, the animals were euthanized with Euthasol. 

### 2.2. Experimental Design

The first study was designed to test if a difference existed in the outcomes of DCD kidneys perfused on a LifePort or an RM3 machine. Eight dogs each donated one kidney to the ORS and one kidney to the Waters pump group in a paired experimental design. Seven dogs in each group completed the study as two animals were euthanized 3 days after surgery due to severe giardiasis and their data were not included in the study. A third group was used to test the effects of pulsatility per se on preservation of DCD kidneys by using the LifePort in constant pressure mode (30 mm Hg mean) with no pulsations and comparing those results to the group run on the LifePort in pulsatile mode (30 mm Hg mean). 

### 2.3. Statistical Analysis

All data were tested for normality of distribution and found to be normal. Descriptive statistics (mean, standard deviation, count, and standard error of the mean) were determined with statistical software (GraphPad, V4.1). Differences in the means between groups were analyzed by GraphPad software using an unpaired *t*-test (2 groups) or one-way ANOVA with Dunnett's multiple comparison test (>2 groups) using an *α* = 0.05. Fisher's Exact test was used for survival ratios.

## 3. Results

**Figure 1:**
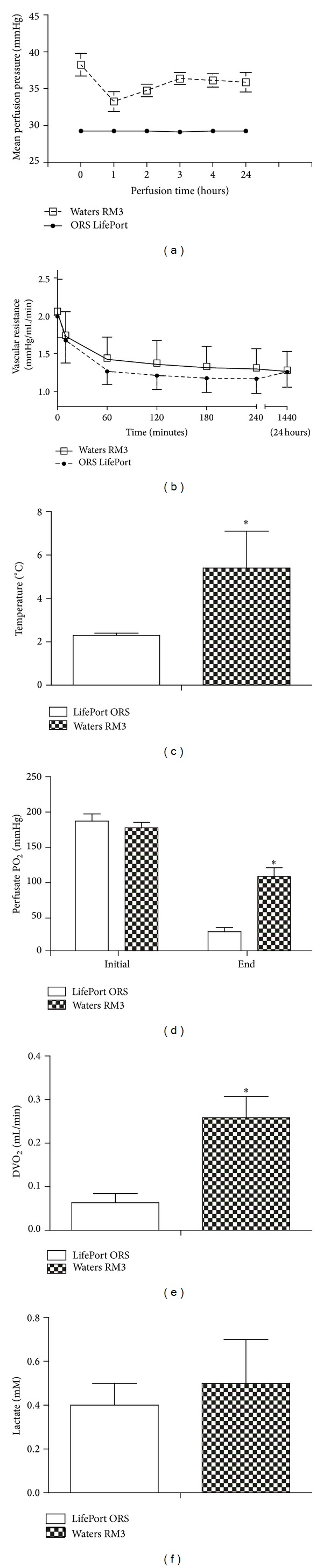
Machine perfusion characteristics of DCD kidneys pumped on a Waters RM3 or an ORS LifePort device for 24 hours. Mean perfusion pressure (a), vascular resistance during perfusion (b), average perfusate temperature (c), initial and end perfusate pO_2_ values (d), end of perfusion delivery of oxygen (DVO_2_) (e), and end of perfusion perfusate lactate concentrations (f) values are mean ± S.D., *P < 0.05 relative to LifePort, *n* = 8 per group.

Machine perfusion parameters over the 24-hour hypothermic perfusion period for kidneys in both machine groups were similar. The mean perfusion pressure was significantly higher by about 6 mm Hg in the Waters group compared to the ORS group (Figure 1(a)). The perfusate flows were also proportionally higher in the Waters perfused kidneys such that the vascular resistance in both groups was the same over the 24-hour perfusion preservation period (Figure 1(b)). 

Metabolic characteristics during machine perfusion were also compared (Figures 1(c)–1(f)). The temperature of operation of the Waters machine was significantly higher than the ORS LifePort (5.5°C versus 2.2°C, resp.). While the metabolic demand was slightly higher in the warmer Waters machine, the delivery of oxygen was significantly higher than LifePort too. Specifically, the perfusate pO_2_ started off identical in both machines at the start of perfusion preservation, but 24 hours later, the perfusate from the Waters machine had a significantly higher pO_2_. Since the arterial inflow rates were similar, the higher arterial inflow pO_2_ in Waters translates into a significantly higher delivery of oxygen to the kidney (DVO_2_) compared to the ORS LifePort. In spite of the lower inflow oxygen tension in the LifePort at the end of perfusion, the lactate concentrations in the perfusate were not different (Figure 1(f)). Perfusate electrolyte and pH were the same in both groups. 

**Figure 2:**
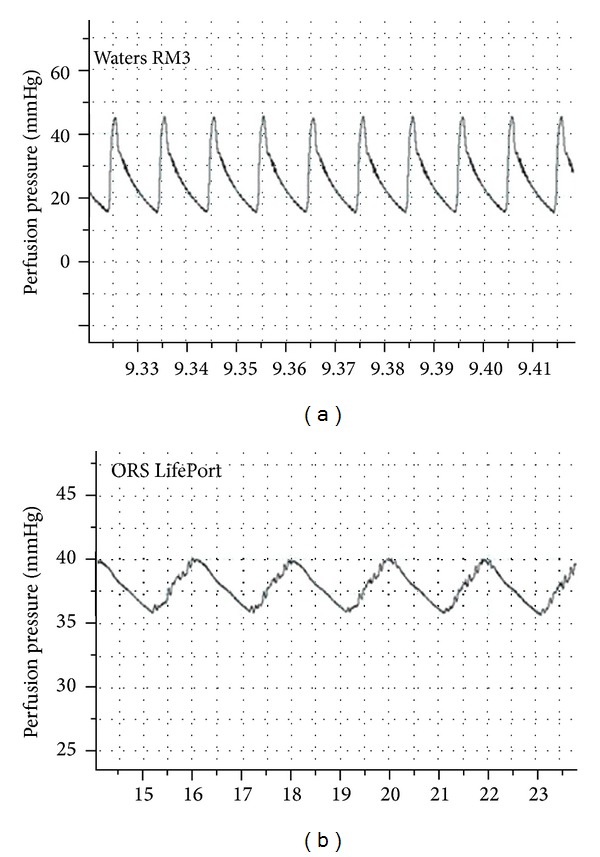
Samples of arterial pressure waveforms from a Waters RM3 and an ORS Lifeport during perfusion preservation.

An analysis of the arterial pressure waveform of both machines revealed some significant differences. While both devices produce a pulsatile waveform, there are some interesting qualitative differences. A pressure transducer connected to the arterial side of the perfusion pump recorded real-time changes in arterial pressure in both Waters and ORS device (Figure 2). Both machines produced pulsatile flow but the LifePort pressure waveform was clearly wider, shorter, and exhibiting a smaller pulse pressure, relative to the RM3 arterial pressure waveform.

**Figure 3:**
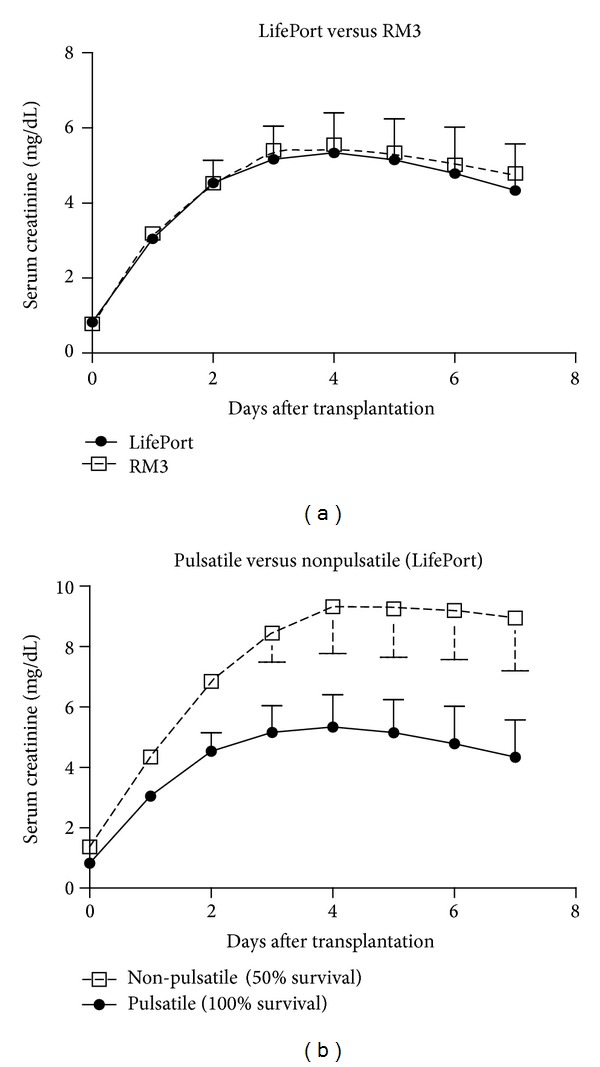
Daily serum creatinine values after kidney autotransplantation after DCD donation. Kidneys were perfused for 24 hours using the LifePort (Open Squares) or the RM3 (Solid Circles). Values are mean ± S.D. and represent 8 dogs per group (Figure 3(a)). Similar data (Figure 3(b)) are shown for a LifePort operated under either constant pressure (nonpulsatile flow) or pulsatile flow conditions (both at 30 mm Hg). The values are statistically different between the first 3 corresponding post-operative days. The survival in the pulsatile and nonpulsatile groups were 100% (8/8) and 50% (2/4), respectively (*P* = 0.09).

Posttransplant renal function after 24 hours of perfusion preservation was assessed for 7 days after transplantation to index the degree of renal preservation injury suffered by each group of kidneys from DCD donors. True DGF cannot be assessed in this model, so the model is calibrated to cause maximal renal injury without causing death from renal failure. The results shown in Figure 3(a) indicate that the serum creatinine values after transplantation were essentially identical in both machine groups. The injury produced by this model, relative to a non-DCD injury, and the efficacy of machine perfusion, relative to cold storage, has been previously characterized in detail by our group [4]. While there was no difference in outcomes between the two perfusion machines, the presence of a pulsatile pump duty cycle was important. Specifically, pulsatile perfusion significantly improved outcomes of DCD kidneys after transplantation since a significant potentiation of preservation injury was observed in kidneys from DCD donors that were perfused on a LifePort under constant pressure conditions (nonpulsatile mode), relative to perfusion on the LifePort in pulsatile mode (Figure 3(b)).

## 4. Discussion

Perfusion preservation of kidneys is becoming more common, especially since the rates of ECD and DCD donation have significantly increased. This upward pressure has led to more careful analysis of available machine perfusion devices regarding their differences, similarities, and capabilities under today's donation conditions. The two most popular devices used internationally today are the Waters RM3 and the ORS LifePort. These devices have significant differences in design and operation, but this study has clearly demonstrated that both machines perform equally for the hypothermic perfusion preservation of DCD kidneys, as indexed by the evaluation of posttransplant renal function in a proven dog renal autotransplant model. This model was used to accurately test UW solution 20 years ago and it correctly predicted the clinical response for human organs so its use for this study is appropriate and valid for predicting the clinical effects of these two machines under simulated DCD conditions.

The “Mini Belzer Unit” kidney perfusion machine was the first portable kidney perfusion machine used by the University of Wisconsin for routine preservation of kidneys for transplantation beginning in 1972 and used up until 2008. The commercial device later produced by Waters copied the general design and perfusion drive of the Belzer Machine, which consisted of an electric motor driven pump that cyclically squeezed perfusate out of a compressible bladder between two moving plates. These pumps contained one-way valves, which mimicked the heart and produced truly physiological pulsatile arterial pressure waveforms that look remarkably like an arterial pressure waveform in a human. These waveforms even have a clearly identifiable dicrotic notch, representing the closure of the outflow valve during the end of pump systole. The success of the Belzer machines and the similar Waters machines have been attributed to this specific pump duty cycle and the physiological pressure waveforms that they produce in addition to active oxygenation of the perfusate. However, this study shows similar outcomes with the ORS LifePort, which produced a more attenuated pulsatile flow and pressure waveform and has no means of perfusate oxygenation. The LifePort arterial pressure waveform has a significantly smaller pulse pressure and few physiological characteristics like a dicrotic notch or a diastolic decay. In constant pressure mode (no pulsatility), the ORS LifePort performs significantly worse compared to the pulsatile mode under near identical conditions (Figure 3(b)). Thus, it appears from these studies that pulsatile flow per se is an important and necessary attribute for good perfusion preservation of DCD kidneys but the magnitude or quality of the pulsations is less important.

The utility of machine perfusion preservation for DCD kidneys was not measured in this study but was assumed. The goal of this study was to determine which of two commonly used clinical machines were more useful for preserving DCD kidneys but not if perfusion is better than cold storage. The usefulness of hypothermic machine perfusion per se for DCD kidneys over simple cold storage was first demonstrated by Lindell et al., in a preclinical study in 2005 [4] and later for DCD patients [9] and for non-DCD patients [7, 8]. Therefore, only a simple experimental design consisting of DCD kidneys perfused on either a LifePort or an RM3 was needed to complete the objectives of the study.

The need for oxygenation of the kidney perfusate in clinical devices has been debated. Some perfusionists claim that oxygenation of the perfusate is necessary, either from room air or oxygen facilitated by an extracorporeal membrane oxygenator or from passive equilibration with the atmosphere. Many also believe that this is critical for successfully preserving injured kidneys from DCD donors. The ORS LifePort has no provision for either active or passive oxygenation of the perfusate since it was designed for portability. However, it performs as an RM3 or a Belzer machine, which both have active oxygenation. This study suggests that physiological levels of oxygenation in the perfusate are not necessary for good perfusion preservation of DCD kidneys since there were no differences between the two machines, which had significantly different perfusate pO_2_ levels at the end of perfusion. However, the ORS was run at a cooler temperature, which reduced oxygen demand compared to the RM3. Neither pump in this study had a limiting oxygen delivery since the ends of perfusion lactate levels were the same in each group and were low, suggesting that anaerobic glucose fermentation was not occurring secondary to inadequate oxygen delivery. This even occurred in the ORS LifePort that had perfusate pO_2_ levels around 30 mm Hg after 24 hours of perfusion preservation. Thus, at profound hypothermic temperatures, active or passive perfusate oxygenation is not necessary, even for DCD kidneys. While the Waters RM3 has a lot of oxygen delivery reserve capacity because of its active oxygen transfer properties, the ORS LifePort may be close to “running on empty” after 24 hours. Therefore, longer perfusion times past 24 hours for DCD kidneys are not recommended in the LifePort without some form of passive or active oxygen transfer. These features were avoided in the LifePort design to produce portability and to decrease heat transfer, which are important attributes of the LifePort. Since human kidneys are much larger and have higher absolute oxygen consumption rates under these conditions, relative to our small Beagle dog kidneys, there should be more concern of limiting oxygen delivery when the device is used in humans for more than 24 hours. 

Sufficient oxygen delivery to kidneys perfused at 4–6°C with crystalloid preservation solutions is not an issue since the slow metabolic rate of the kidneys at such profound hypothermic temperatures reduces the metabolic demand to about 3% of baseline rates [10]. At this low level of metabolism, slow flow rates of water based solutions, either equilibrated with room air or oxygen (RM3) or not equilibrated at all (LifePort), produce sufficient oxygen delivery in both machines. The lack of lactate accumulation under these conditions proves this point. However, if higher perfusion temperatures or longer duration of perfusion is attempted with the LifePort, then the DVO_2_ may need to increase, either through higher flow rates, a higher O_2_ carrying capacity (carriers), higher O_2_ concentration of the solution, or combinations of these. An additional source of oxygen will be required to facilitate oxygen transfer to the solution phase. Therefore, the LifePort would need to adapt some form of active or passive oxygen delivery in order to meet these demands whereas the RM3 already has some of this capacity. The new version of LifePort has an external oxygenation option.

The current study modeled clinical DCD kidney donation by inducing in situ warm renal ischemia before recovery and preservation in large animals, which does not completely model the conditions in clinical DCD donors. The question is whether any modeling shortcomings represent significant deviation from the clinical condition and whether these differences prevent a correct analysis of the results. The ischemia induced in situ in this study, while producing a clinically relevant degree of warm ischemia, is produced under conditions that differ in clinical DCD recovery. Specifically, the physiological, biochemical, and immunological changes associated with trauma and agonal cardiac death in DCD patients before organ recovery are missing in our animal model and may influence preservation injury. However, the model was the same for both groups, so any missing effect would be controlled and treated equally between the two groups. Therefore, any real differences between the model and real clinical experiences should not alter the conclusions of the study, which were based on the relative comparisons between the two machine perfusion groups. Finally, any physiological, biochemical, hormonal, or immunological adjustments to cardiac death that were missing from the current DCD model may not have produced a measurable difference in the outcomes anyway since similar adjustments, due to the agonal phase after explosive brain death, are not observable in the same large animal kidney transplant model [11].

Another potential problem with this model is the severity of the induced renal failure. Specifically, uncontrolled DCD kidneys in the clinic will often cause DGF and require renal replacement therapy with dialysis until the graft begins to regain function over the coming weeks. Then, the graft may be at higher risk for developing chronic allograft nephropathy (CAN) months later. Our model does not induce renal injury severe enough to cause clinical DGF since the animals cannot be placed on dialysis. Therefore, we calibrate the severity to not cause animal death and rely on renal graft function after transplantation as a measured outcome of preservation injury. Furthermore, since our model is an autotransplant, we miss any long-term nephropathy because the allogeneic immunological background is lost, which is probably essential for the development of CAN [12]. However, this model is still useful since renal autograft function measured after the first week of transplantation is proportional and predictive of preservation injury in the graft [13].

In conclusion, this study has determined that DCD kidneys can be effectively machine perfused for 24 hours on either an ORS LifePort or a Waters RM3 with equal outcomes, provided that the LifePort is used in pulsatile mode. Each device has both positive and limiting attributes that must be considered, especially when using them under unconventional conditions.
